# e-SCP-ECG^+^ Protocol: An Expansion on SCP-ECG Protocol for Health Telemonitoring—Pilot Implementation

**DOI:** 10.1155/2010/137201

**Published:** 2010-06-07

**Authors:** George J. Mandellos, Michael N. Koukias, Ioannis St. Styliadis, Dimitrios K. Lymberopoulos

**Affiliations:** ^1^Wire Communication Laboratory, Electrical & Computer Engineering Department, University of Patras, Rion GR-26504, Greece; ^2^Cardiology Department, General Hospital of Aegion, Aegion GR-25100, Greece

## Abstract

Standard Communication Protocol for Computer-assisted Electrocardiography (SCP-ECG) provides standardized communication among different ECG devices and medical information systems. This paper extends the use of this protocol in order to be included in health monitoring systems. It introduces new sections into SCP-ECG structure for transferring data for positioning, allergies, and five additional biosignals: noninvasive blood pressure (NiBP), body temperature (Temp), Carbon dioxide (CO_2_), blood oxygen saturation (SPO_2_), and pulse rate. It also introduces new tags in existing sections for transferring comprehensive demographic data. The proposed enhanced version is referred to as e-SCP-ECG^+^ protocol. This paper also considers the pilot implementation of the new protocol as a software component in a Health Telemonitoring System.

## 1. Introduction

Nowadays, the health management of the elderly and less able or those with chronic health problems tends to guarantee their quality of life. It is suggested that there should be continuous monitoring of their daily activities and the evaluation of their vital operations and the context parameters that affect them. The long-term monitoring of such factors should indicate all changes or trends in an individuals' health status and influence the response and management of their diseases [1]. Hence, for example, a patient's living and working conditions in correlation with possible allergies may aggravate his health or considerably affect his medication.

Current efforts for the accomplishment of this objective tend towards the creation of ubiquitous Health Telemonitoring Systems (HTSs) that enable remote observation of an individual and decision-making in case of a health crisis (e.g., with the intervention of health experts). The HTSs allow these persons to move and act without constraints, according to their abilities. They may also be applied efficiently for persons with different profiles, for example, epileptic, diabetic, asthmatic, and phobic.

Hence, the HTSs should be based upon open and flexible architectures allowing the integration of various vital and context information. From the architectural point of view, they usually include two core entities. The first is a Body Area Network (BAN) acquiring biosignal information, such as Electrocardiogram (ECG), noninvasive blood pressure (NiBP), body temperature (Temp), and pulses. The second entity acquires context awareness, positioning and environment information, such as humidity and temperature [2].

The integration of the whole acquired vital and context data into the structure of a single file or message is critical for the efficient operation of the HTSs within client-server or peer-to-peer (P2P) applications [3–5]. In this way, both the total transactions of the final user and uncertainty in the affirmation of an accurate correct decision are minimized.

Note that some standards organizations have already defined file formats and protocols integrating different types of medical information. They usually include trivial patient elements, such as code, name, surname, weight, and height. These structures integrate primarily ECG and electroencephalogram (EEG) biosignals and secondarily the NiBP, Temp, blood Oxygen Saturation (SPO_2_), Carbon dioxide (CO_2_), pulse rate, and so forth. For example, the SCP-ECG integrates ECG, TEMP, and NiBP, the EDF+ integrates EEG, ECG, and CO_2_, while the CEN File Exchange Format is the only structure that integrates more biosignal types. The characteristics and the performance of these protocols are extensively analyzed in [6–8].

It is obvious that none of these protocols is able to support by itself the full functionality of the HTSs. Two or more protocols should be used concurrently, thus introducing a high degree of complexity.

The main aim of this work is to analyze the capabilities of the existing medical data protocols and to propose respective expansions for them, in order to support the full functionality of the HTSs. This analysis has led us to propose an extension of the SCP-ECG protocol; the expanded protocol is thereafter referred as e-SCP-ECG**^+^** protocol.

The structure of e-SCP-ECG**^+^** protocol includes sections for vital, context aware, and patient-centric data. The sections for vital signs comprise at least six biosignals (ECG, NiBP, SPO_2_, Temp, CO_2_, and Pulse Rate) as well as plethysmographic (PLE) data for SPO_2_. The context aware section comprises at the very least the geolocation data and altitude. The patient-centric information section incorporates data about allergies, blood group, environmental elements (residence, work, etc.), and personal constraints (e.g., interdiction of blood-transfusion for religious reasons).

This paper also demonstrates the pilot implementation of an HTS that makes use of the e-SCP-ECG**^+^** protocol. Earlier versions of the e-SCP-ECG**^+^** protocol have been already tested in telemedicine projects [4, 5]. 

Section 2 presents the state of the art in existing medical standards and also the reason we choose to extend the SCP-ECG protocol. Section 3 presents the extensions included in the e-SCP-ECG**^+^** protocol. Section 4 presents the implementation of a pilot HTS using the e-SCP-ECG**^+^** protocol. In Section 5 the authors discuss the advantages of the e-SCP-ECG**^+^** protocol and the pilot real world operation of the implemented HTS used to evaluate the new protocol. Finally, a conclusion is reached in Section 6.

## 2. Existing Medical Data Standards in Health Monitoring

### 2.1. State of the Art in Existing Medical Data Standards

This chapter analyses the characteristics of the existing standards employed for the organization, management, and distribution of biosignals and other medical information. 

*SCP-ECG (CEN/ENC 1064 Standard Communication Protocol for computer assisted Electrocardiography)* [9, 10] is a standard data format established by the European Standard Committee (CEN) for ECG recordings. It defines the patient's ECG data structure, the basic demographics format, and also rules for data interchange between digital ECGs and computer systems. It can handle binary signals and annotations in a number of defined sections as they are obtained in different tests with ECG recordings. These tests include short-term and long-term ECG recordings, stress tests with ECG, and also angiography with ECG. In addition, it can handle data compression using known algorithms. It is a recommendable alternative to ECG databases.

*DICOM (Digital Imaging Communication in Medicine) supplement 30* [11] covers the waveform acquisition within the imaging context. It is specifically meant to address waveform acquisitions to be analyzed with other data transferred and managed using the DICOM protocol. It allows the addition of waveform data to that context with minimal incremental cost. Further, it leverages the DICOM persistent object capability for maintaining referential relationships to other data collected in a multimodality environment, including references necessary for multimodality synchronization.

*HL7 (Health level 7)* is an international organization developing healthcare standards for clinical and administrative data. The HL7-annotated ECG (HL7 aECG) [12] was developed by the HL7-Regulated Clinical Research Information Management Technical Committee using the draft-annotated ECG nomenclature developed by IEEE 1073. 

*ISHENE Standard Output Format for Digital Holter Data* [13] is a single file structurally organized in a header followed by a (larger) data block containing all stored ECG digital samples.

*EDF (European Data Format)* [14] is a simple and flexible format for exchange and storage of multichannel biological and physical signals. The signals can have any physical dimensions and sampling frequencies. The EDF file has an ASCII header containing mainly patient and time identification, the number of signals, and the technical characteristics such as the main dimension, calibration values, and sampling frequency of each signal. The header is followed by subsequent data records. The duration of the data records is specified in the ASCII header. The EDF+ file can also contain interrupted recordings, annotations, stimuli, and events.

*CEN/FEF (File Exchange Format for Vital Signs)* [15] incorporates data items coming mainly from intensive care units, anaesthesia departments, and clinical laboratories including neurology. The FEF biosignal files consist of sections. Each section begins with a tag for section identification purposes, followed by a length field indicating the length of the section and the actual data. The demographics section contains information about the recorded patient, whereas the healthcare provider section stores basic textual data of the healthcare institution and personnel that collected the data. A section concerning the medical device system presentation contains a structured description of the devices (one or many) that participated in the data collection. There is also an optional manufacturer-specific section. The FEF definition process is a serious attempt to unify biosignal and related measurement offline storage needs for both the various electrophysiological laboratories and intensive care/anaesthesia departments.

*IEEE 1073* is a comprehensive standard for electronic signal data communication between medical devices and bedside monitoring devices [16]. It is designed specifically for acute care, with requirements such as the ability to handle frequent network reconfiguration, the plug and play operation, the robust and reliable communications for a safety critical application, and the association between a device and a specific patient [17]. This protocol is specialized at the medical device level, and it is not always easy to apply.

*MFER (Medical waveform description Format Encoding Rule)* [18] is a standard developed by the Japanese standard organization, specialized for waveforms such as ECG and EEG. It is only specialized in medical waveforms. For encoding information other than medical waveforms, it recommends the usage of another format such as the HL7, DICOM, or IEEE 1073.

*ASTM 1467* [19] is the only standard for neurophysiology supported by the American standard body. It offers support for EEGs, polysomnograms (PSGs), evoked potentials (EPs), electromyograms (EMGs), and so forth. It is also suitable for ECGs.

*EBS (Extensible Biosignal Format)* [20] is a simple binary file format for storing multichannel time-series recordings and associated metadata. It was used primarily for handling EEG, MEG, and ECoG recordings from human brains. It can also handle the patient's or subject's name, identifier, date of birth, sex, and various other data relative to the acquisition process.

### 2.2. Why We Adopt the Solution of Extending SCP-ECG Protocol

During the investigation period of the above protocols in order to find a protocol that meets our needs and also is easily expandable and independent from “parent” protocols (e.g., DICOM and supplement 30), we studied and analyzed the aforementioned protocols and also some corresponding papers like [6–8]. The analysis has demonstrated that two of these are closer to our needs, the CEN/FEF and the SCP-ECG. The CEN/FEF protocol, although covering a wide area of biosignals, has increased complexity of implementation. This protocol, moreover, has rare implementations [6]. On the other hand, the SCP-ECG protocol is well defined and structured, provides the widest expansion capabilities, and is supported by many major manufacturers of ECG equipment [6]. More precisely, it incorporates a patient's ECG data structure, an elementary demographics format, and rules for interchanging data between digital ECG carts and hosts that, respectively, acquire and store the ECG data. 

All this information is included in an SCP-ECG formatted file that provides twelve (12) sections dedicated per information category (Table 1). Despite the protocol being designed to handle data on ECG measurements, these sections handle a significant amount of information. This file handles the minimum set of patient demographic data, a pair of values for the NiBP (low and high), and a value for heart pulse rate. It has also the capability to handle ECG data acquired using a different sampling rate, limited to a few minutes. Finally, it includes several sections for the handling of manufacturer-specific content that can adequately handle various types of information through continuous health monitoring applications.

## 3. The Proposed e-SCP-ECG^+^ Protocol

The e-SCP-ECG**^+^** [21] protocol extends the SCP-ECG protocol in order to handle more information about patients and their vital signs than those asked by doctors during various telemedicine projects in the past. 

So, the e-SCP-ECG**^+^** [21] protocol extends the existing Section-1 of the SCP-ECG with new tags for extra demographic related data and data reference to the equipment. Moreover, it extends the file structure of the SCP-ECG defining new sections (Section-200 to Section-207) for handling additional data, such as extra biosignals data, and the allergies, which are required for patient's health monitoring.

### 3.1. New Tags in Section-1

Section-1 is designed to transmit patient demographic data, as well as technician, physician, and equipment identification data. Flexibility is achieved by organizing different information within different successive recorded header fields. All header fields have a similar structure that consists of three parts: a “tag” (one byte) that indicates the contents of the parameter field, a “length” (two bytes) containing the length of the field value, and a “value” (zero to 65 Kbytes) containing the actual parameter data.

The protocol supports 255 (0 to 254) different header field types. The first 36 types (tag 0 to 35) and the last one (tag 255) are already used by the SCP-ECG protocol (Table 2), and 55 field types (tags 200 to 254) are reserved for use by any individual manufacturer. In this work, we propose the use of 17 field types (tags 200 to 216) for the extra needs of the e-SCP-ECG**^+^** protocol. 

Table 3 depicts the proposed specification of the newly defined parameters included within the field types with tags 200 to 216. These fields are defined as optional. In some cases, selected fields may be labelled as “unethical”, and the patient's agreement is critical in order for them to be filled in. For example, the “patient religion” field should be filled in only special cases, in order to indicate nonmedical restrictions affecting the applied medical treatment, for example, blood transfusion that is prohibited in some religions. 

### 3.2. New Sections

The SCP-ECG protocol currently defines section ID numbers 0 through 11 in its structure, reserves section numbers 12 to 127, as well as numbers above 1023, for future use, and leaves numbers 128 through 1023 for manufacturer-specific sections. The e-SCP-ECG**^+^** protocol assigns and uses the following extra eight (8) new sections:

- Section-200 for SPO_2_ and arterial pulse rate data,
- Section-201 for Temp data,
- Section-202 for CO_2_ data,
- Section-203 for NiBP data (systolic—diastolic) and Pulse Rate data,
- Section-204 for allergy information,
- Section-205 for plethysmographic (PLE) data,
- Section-206 as an extension of Section 6 for long-length/interrupted ECG data,
- Section-207 for user's geolocation using Global Positioning System (GPS) data.

All the sections of the e-SCP-ECG**^+^** protocol adopt the general sections format of the SCP-ECG protocol constituted of two parts, the section Identification Header and the section Data Part [9]. The section Identification Header part is used without any modification. Below the structure of the Data Part (DP) of the eight (8) new sections is analyzed.

**Figure 1:**
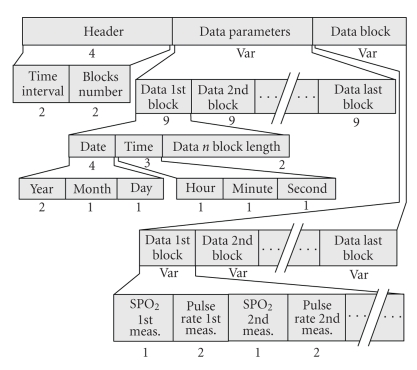
Overview of the data part holding the SPO_2_ and pulse rate data (Section-200).

The *Section-200 DP* handles pairs of SPO_2_ and Pulse Rate data samples acquired either using a permanent rhythm or asynchronously. It contains three parts (Figure 1). The “DP Header” keeps data on the sampling rate (time interval) and the number of data blocks collected. The “Data Parameters” determine parameters for each data block (date, time, and block length). The “Data Block” keeps successive recordings of the periodically acquired pairs of SPO_2_—Pulse Rate values. 

**Figure 2:**
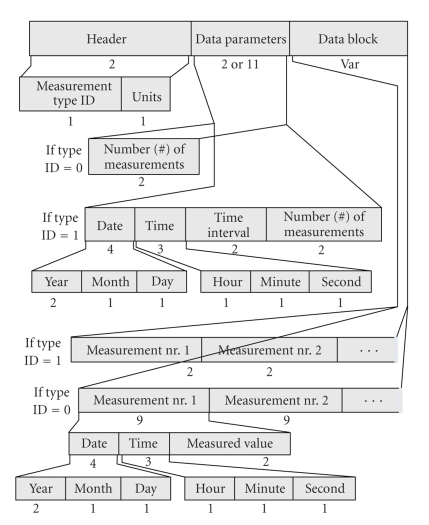
Overview of the data part holding the temperature data (Section-201).

The *Section-201 DP* handles the Temp data samples acquired by using a permanent rhythm (measurement type (mt) equal to “1” (mt = 1)) or asynchronously (mt = 0). It consists of the “DP Header”, which contains two parts (“mt” and “Units”), the “Data Parameters” and the “Data Block” (Figure 2). If mt = 1, “Data Parameters” records Date, Time, time interval, and number (#) of measurements. If mt = 0, “Data Parameters” record only the number (#) of measurements. The “Data Block” records the captured measurements. If mt = 1, “Data Block” keeps the successive recordings of the periodically acquired Temp values. If mt = 0, “Data Block” keeps successive recordings of distinct Temp measurements (Date, Time and Temp value).

**Figure 3:**
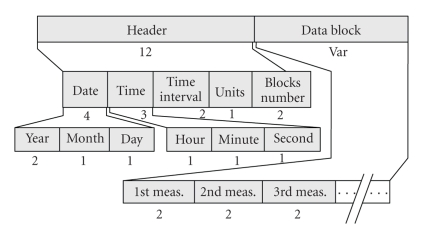
Overview of the data part holding the carbon dioxide data (Section-202).

The *Section-202 DP* handles the CO_2_ data. It contains two parts (Figure 3). The “DP Header” handles parameters of captured data (date, time, time interval, CO_2_ units, and the number (#) of measurements). The “Data Block” keeps successive recordings of the periodically acquired CO_2_ values.

**Figure 4:**
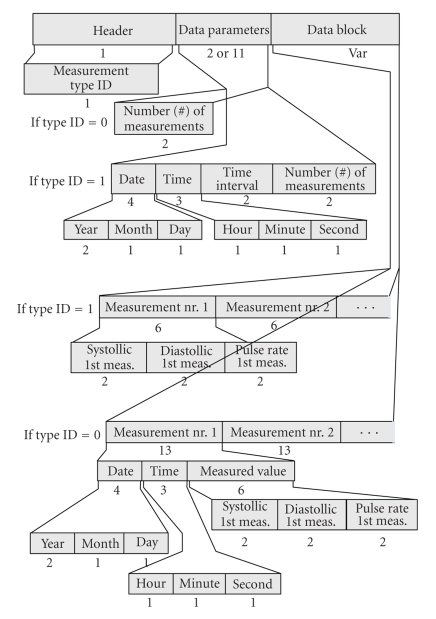
Overview of the data part holding the systolic-diastolic blood pressure data (Section-203).

The *Section-203 DP* handles triples of systolic-NiBP, diastolic-NiBP, and Pulse Rate data samples acquired either through a permanent rhythm (mt = 1) or asynchronously (mt = 0). It consists of the “DP Header”, which contains the “mt”, the “Data Parameters”, and the “Data Block” (Figure 4). If mt = 1, “Data Parameters” records Date, Time, time interval, and number (#) of measurements. If mt = 0, “Data Parameters” record only the number (#) of measurements. The “Data Block” records the captured triples of measurements. If mt = 1, “Data Block” keeps the successive recordings of the periodically acquired values. If mt = 0, “Data Block” keeps successive recordings of distinct measurements (Date, Time, and values).

**Figure 5:**
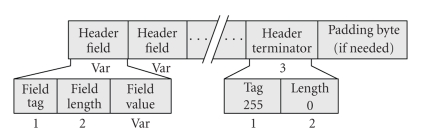
Overview of the data part holding the allergy data (Section-204).

The *Section-204 DP* handles the data of five (5) allergies (rhinitis, asthma, medical allergy or drug allergy, food allergy, and other allergy). It has the same structure as Section-1, containing six (6) header fields, a header terminator, and a padding byte (Figure 5). Each header field concerns of a specific type of allergy and consists of the tag, length, and value fields. Table 4 shows the specification of the defined parameters. 

**Figure 6:**
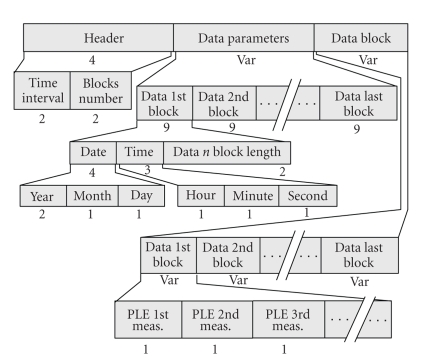
Overview of the data part holding the plethysmographic data (Section-205).

The *Section-205 DP* handles the plethysmographic (PLE) data samples acquired either through using a permanent rhythm or asynchronously. It contains three parts (Figure 6). The “DP Header” keeps data on the sampling rate (time interval) and the number of data blocks collected. The “Data Parameters” determine parameters for each data block (date, time, and block length). The “Data Block” keeps successive recordings of the periodically acquired PLE values. 

**Figure 7:**
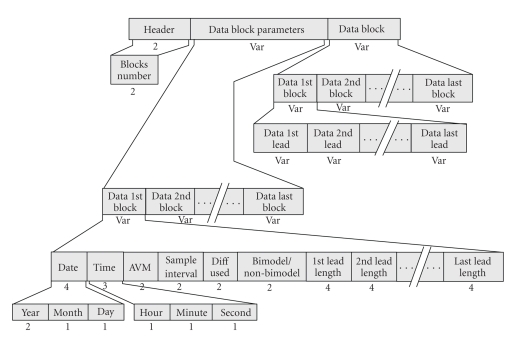
Overview of the data part holding the extended ECG data (Section-206).

The *Section-206 DP* keeps long-length ECG recordings or interrupted recordings. It is an extension of Section-6 of SCP-ECG, which handles the ECG waveform data. Section-206 contains three parts (Figure 7). The “DP Header” keeps data on the number (#) of data blocks collected. The “Data Parameters” determine parameters for each data block (date, time AVM, Sample Interval, Used compression (Differential, Bimodal), 1st lead length, 2nd lead length,…, last lead length). The “Data Block” keeps successive recordings of the 1st lead-data, 2nd lead-data,…, and last lead-data. The term “lead-data” corresponds to the total acquired measurements of this lead.

**Figure 8:**
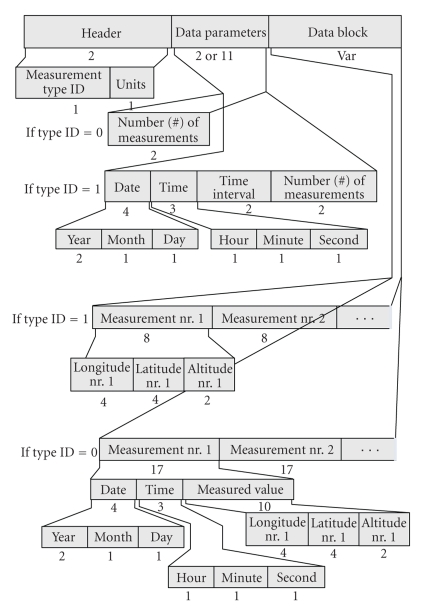
Overview of the data part holding the GPS data (Section-207).

The *Section-207 DP* handles positioning data (triples of Longitude, Latitude, and altitude) acquired in accordance with NMEA 0183 protocol [22]. The positioning data can be acquired either through the use of a permanent rhythm (mt = 1) or asynchronously (mt = 0). It consists of the “DP Header”, which contains the “mt”, the “Data Parameters”, and the “Data Block” (Figure 8). If mt = 1, “Data Parameters” records Date, Time, time interval and number (#) of measurements. If mt = 0, “Data Parameters” record only the number (#) of measurements. The “Data Block” records the captured triples of measurements. If mt = 1, “Data Block” keeps the successive recordings of the periodically acquired triples of values. If mt = 0, “Data Block” keeps successive recordings of distinct measurements (Date, Time, and triples of values).

## 4. Implementation of a Pilot HTS Using the e-SCP ECG^+^ Protocol

### 4.1. HTS Architecture

**Figure 9:**
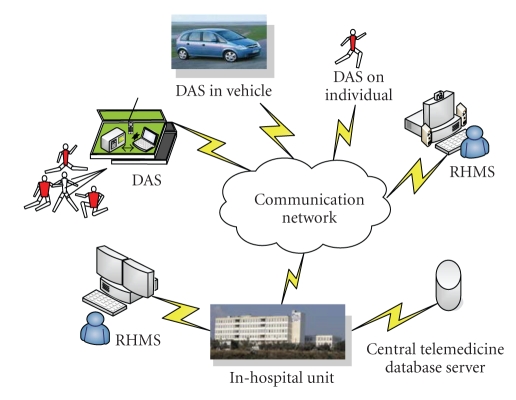
Structure of the designed Health Telemonitoring System.

This part demonstrates the pilot implementation of the e-SCP-ECG**^+^** protocol as a software component. This component is integrated and evaluated in an HTS for individuals suffering from heart problems (Figure 9). The individuals' vital signs are acquired, archived, manipulated, and processed by three types of entities.

**(i) Data Acquisition System (DAS)**

**Figure 10:**
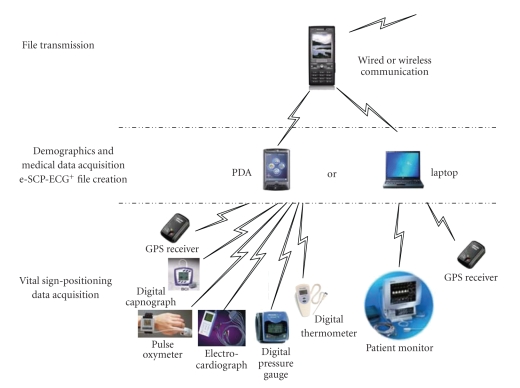
Overview of data acquisition, file preparation, and transmission.

We use a wearable and a conventional portable DAS (Figure 10). The wearable DAS consists of vital sign measurement sensors (ECG, NiBP, SPO_2_, Pulse Rate, Temp, and PLE) able to communicate via Bluetooth, a GPS receiver (RoyalTek RBT-1000), and a Personal Digital Assistant (PDA) device including Mobile ADSL capabilities. The wearable DAS is handled directly by the individual himself. The conventional portable DAS consists of a medical monitor (MM) (Criticare's Poet Plus 8100) acquiring vital signals (ECG, NiBP, SPO_2_, Pulse Rate, Temp, PLE, and CO_2_), a GPS receiver (RoyalTek RBT-1000), and a laptop (HP/Compaq nw8440) device including Mobile ADSL capabilities. The portable DAS is handled by a technician or a doctor and is already tested on another telemedicine system whose general architecture has been analyzed in [23] (without the GPS capability). The demographic and allergy data are introduced manually into PDA and laptop devices. The collected information in these devices is automatically organized in e-SCP-ECG**^+^** files for local storage and forwarded to a remote expert's site for monitoring.

**(ii) Remote Health Monitoring System (RHMS) Covers the Expert's Site**

It consists of a PC including a special application for opening e-SCP-ECG**^+^** files and modifying, storing, presentation, and processing of the acquired data. It also includes procedures for comparing old and new measurements.

**(iii) Storage Unit (SU) For Temporary Storage and Archiving of the Received Files**

The DASs in the pilot HTS were used for the remote monitoring of twenty-seven individuals. The monitoring of these individuals was performed by two RHMSs, located one in the Cardiology Department of Aegion General Hospital and the other at the doctor's private site. This department works as the Telemonitoring Centre (TC), also including the SU. The DASs communicate with RHMS using public Fixed and Mobile ADSL.

### 4.2. Analysis of the Health Monitoring Process

The DASs send the e-SCP-ECG**^+^** files to the RHMSs through the SU. The SU has the responsibility to keep the whole body of information permanently in dedicated spaces. This Client/Server (C/S) scheme permits centralized control of the monitoring process, allowing efficient switching of the received files among different RHMSs. The monitoring process is performed by experts operating the RHMSs. The switching capability facilitates the doctors' mobility and the use of different RHMSs without losing contact with the total archives. The switching request in this implementation is initiated by one RHMS user and contains the receiver RHMS ID.

The monitoring process employed is distinguished by a basic and an emergency operational mode. In both modes, the monitoring process requires real-time recording of the individual's vital sign, context, and geolocation data. The recorded data are included within one file and sent to the RHMS.

The basic mode is applied during the detection of normal health conditions of the individual. The type and the recording period of the medical data are directly related to the individual's health condition (status); this status is supposed by the doctors applying medical criteria. The basic mode distinguishes recording periods of 1, 5, 15, 30 minutes as well as 1, 2, 3, 6, 12, or 24 hours. Each recording includes one examination per biosignal type. Each ECG examination contains data acquired by all leads during an at least 10 second measurement time duration (depending on doctor's decision), and by using a sampling rate of 500 samples per sec and per lead. Hence, the ECG examination data are approximately (at least) 10 Kbytes per lead.

The emergency mode is applied when an individual's abnormal health conditions are detected. This mode uses an enhanced periodical evaluation of the whole body of acquired data (e.g., short recording period of 30 seconds, continuous recording).

The basic and emergency monitoring processes require the creation and transmission of e-SCP-ECG**^+^** files that include a different amount of data each time. The proposed structure allows the dynamic manipulation of these, data, since the structure of all sections allows several examinations to be successively recorded within a file. Hence, the use of the e-SCP-ECG**^+^** empowers doctors to apply monitoring processes fully tailored to the real needs of the individuals.

The overall communication between the entities is ensured by exchanging control and signaling data. The transition from basic to emergency mode is performed either manually, by the user himself using the emergency button either by the RHMSs user, or automatically if any change is detected in the acquired Temp, Pulse Rate, CO_2_, and SPO_2_ values. In the present implementation, the ECG's abnormalities are detected by the evaluator. Algorithms for the automated detection of these abnormalities have not been used.

### 4.3. Implementation Issues

**Figure 11:**
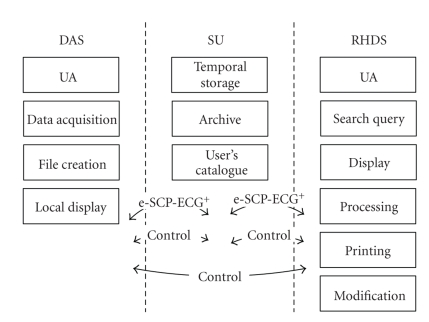
Applications flow diagram.

Figure 11 presents the overall processes employed in the implemented system. The DAS performs User Authentication (UA), Data Acquisition, File Creation, and local data demonstration (Display).

**Figure 12:**
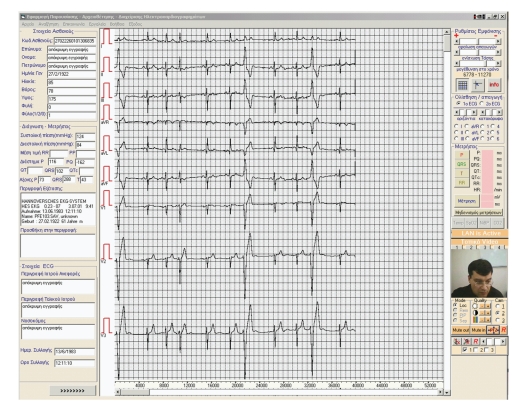
The main screen of the used application on RHMS.

The RHMS performs the UA, Search-Query of archived files, and the Display, Processing, Printing, and Modification of each new file. The modification process allows the user to add diagnostic reports to the received information. The Display processes include dynamic measurement and switching of presentation facilities (change of sensitivity, display speed, zoom of graphical representations) to be applied to ECG signals. The Processing allows comparison of examinations taken on different dates. The comparison is performed directly among arithmetic data or through the superimposition of waveforms and subject evaluation. Figure 12 depicts the main screen of the RHMS.

The SU performs the Temporal Storage and Archiving of the files in an appropriate patient database/file system. The Archiving process handles issues such as reading of files, storing of critical information in the data base, and keeping the original files in a suitably configured tree structure in the disc cluster. The critical information includes the demographic elements, examination date, case subject data, and diagnosis, and it is used for querying purposes any time an expert wants to retrieve an examination. The SU also contains the Users' Catalogue. This User's Catalogue is typically referred to as User Profiles. From the patient's perspective, these profiles could host information such as demographic and personal data as well as crucial information to determine the patient's medical status such as alerting thresholds of physiological parameters [24]. From the medical staff perspective, user profiles host information on personal data, medical specialty, and so forth as well as the list of the patients admitted to each doctor [25] and the availability status.

### 4.4. Security Issues

In the presented implementation, security was provided both during data transmission and upon each user's access.

For the sake of security, the data collected in DAS comprising the e-SCP-ECG**^+^** file were encoded before transmission [1, 26]. The improvement of coding efficiency was achieved by using the Base64 code.

On the other hand, the UA process performs the authentication of the involved (individuals, technicians, physicians, etc.) by establishing the identity of the person and verifying the validity of transferred data. User identification is ensured through a log-in screen that requires a unique id and a unique password for any user to enter. Users already registered in the Catalogue are authorized according to their static role (access privileges, certifications, etc.). These roles are also recorded in the Catalogue to continue a specific navigation inside the data and services provided by the HTS. The access rights of each user, which constitute his individual static role, are defined by his medical specialty. The full data of each user login (user, date, time, etc.) are kept as a user's history.

## 5. Discussion

In order to test the correctness of the proposed extensions, we had to create an SCP-ECG Writer and an SCP-ECG Reader [27, 28]. The SCP-ECG Reader and Writer are software modules entirely written in Visual Basic. During the evaluation of the reader, we used files from the OPENECG portal (established in order to provide help in future protocol implementers) and also from manufacturers such as TAPUZ, QRS, and PROMED PLUS-cardioSCP. Afterwards, we added the e-SCP-ECG**^+^** extensions. The new protocol is designed to be flexible, having an adaptive structure. Handling only the data that characterize each medical incident, it achieves minimum transmission time and also easier management. This attribute makes it ideal for a wide area of applications, such as the transmission of patient data to a reception center, the creation of a medical patient database, and so forth. 

The e-SCP-ECG**^+^** protocol incorporates two fields in its structure in order to set file access rights or to restrict file access. The first field is used in cases where the modification of a file is prohibited. The second one defines the data types accessible to the user. For example, a file can be marked as fully accessible to a specific doctor category/specialty and partially accessible or not accessible to other categories of doctor. This restriction is necessary to ensure that selected experts can access the file contents in order to make the correct diagnosis. The medical specialties and the corresponding restrictions are stored in an appropriate table in DAS's local Database, which is updated through the TC's database server.

The e-SCP-ECG**^+^** protocol ensures data continuity. In many cases, the acquired measurements are not continuous. There are vital signs, such as blood pressure, which can be measured periodically (at defined intervals) or at random time intervals. Also, a measured vital sign can be interrupted for a period in order a special treatment to be applied to the patient. According to the type of acquired data, the e-SCP-ECG**^+^** handles measurements as continuous, distinct, repeated (at specific intervals), or interrupted parts of continuous measurements. 

The e-SCP-ECG**^+^** protocol is designed to overcome the time length limitation of the SCP-ECG protocol. The SCP-ECG protocol handles a limited volume of ECG rhythm data ([9]—Section-6§5.9.3). It defines the length for each lead as a two-byte integer storing for each lead to at most 256 + 256 ∗ 256 = 65792 bytes. Because each measurement is stored as a two-byte integer, the maximum number of allowed measurements for each lead is 65792/2 = 32896. Assuming that any given ECG machine has an acquisition rate of 500 samples per second (no compression is used), the protocol restricts the acquired data to 65.8 seconds. As an improvement, the e-SCP-ECG**^+^** protocol defines the length for each lead as a four-byte integer, each lead therefore holding a maximum of more than 4.311.810.304 values or 2.155.905.152 measurements, which corresponds approximately to 1.200 hours of measurements (with a device acquiring 500 samples per second). 

This implementation targets the evaluation of e-SCP-ECG**^+^** protocol under real world operation. The pilot HTS has been implemented in order to evaluate the e-SCP-ECG**^+^** protocol and was not indented to demonstrate the advantages or the disadvantages of various devices that were used for the collection of vital signs from the human body.

Our prime intention to include all patients who visited the cardiology department of the General Hospital of Aegion during the evaluation period proved impossible because only 60% satisfied the requirements of this study. Their unfamiliarity with the IT systems or with the placement of the sensors used to acquire vital signs was the major problem. The individuals chosen were middle aged (30–60), familiar with IT systems to an acceptable extent, and collaborative. Thus, we ensured the best conditions to evaluate the HTS. All sampled individuals suffer from heart diseases, whereas some of them suffer from hypertension, respiratory, and/or allergies. Table 5 presents the distribution of participants per kind of equipment, disease and age zone.

During a five-month pilot period of HTS operation we aimed to evaluate its reliability. The twenty-seven (27) individuals selected suffer from heart diseases and had prehospital notification. Fourteen (14) individuals used wearable DASs, while the other thirteen (13) used conventional portable DASs. The evaluation of the received data was performed by 3 doctors.

The wearable DASs employed are not embedded in clothes but are composed of discrete devices communicating with the PDA through wireless network. The use of discrete device interfacing groups and a family of sensors (e.g., ECG, CO_2_) is not practical for continuous use due to device volumes, sensor placement, and consequent discomfort. These devices need more care (cleaning, charging, etc.). The gels used in the electrodes dry out after a period of usage, which leads to incremental contact resistance and the subsequent degradation of signal quality. The gels used in the electrodes cause irritation and rushes when used for long periods. The usage of the conventional electrodes requires suitable preparation of the skin if it is hairy. The acquired signals are affected by motion artifacts and the baseline wanders as the electrodes float on the layer of gel.

We also encountered some more problems [29] such as the usage of nail polish which prevents the correct operation of SPO_2_ sensors or cold temperatures or high altitudes, which leads to lower flow of blood in the peripherals resulting in wrong SPO_2_ measurements. These above presented difficulties kept us from achieving long-term monitoring of individuals. Hence, the monitoring process for each individual was limited to a maximum of four hours per day for two or three days per person. Nevertheless, this time is adequate to indicate the overall performance of the e-SCP-ECG**^+^** protocol. The availability of a limited number of devices also prevented us from performing long-term evaluation. 

On the other hand, the conventional portable DASs were utilized in cars during individual transportation from the city of Patras to the city of Aegion, and vice versa. In this case, the difficulties we faced were the bulkiness of the acquisition device and laptop and also the hampering of the wires used to connect the individual to the acquisition device. The length of usage of the devices in this case was equal to the transportation time. 

Before the initial HTS operation, we had to inform the individuals about the system, the placement of the sensors on their body, the operation of the devices, and the way they are charged. 

In order for HTS to be efficient and to overcome possible interrupted transmissions, due to the topology or due to the lack of a High-Speed Packet Access (HSPA) signal, we decided to perform continuous creation and transmission of data snapshots [30] following a predefined interval. Each snapshot was defined to be an e-SCP-ECG**^+^**-formatted file. The interval was selected by the system operator and differed according to each patient's health status, location, and activities. The e-SCP-ECG**^+^** files received by the SU in the TC, after their processing (extraction of critical information), are available to experts. The files concerning the same incident, post diagnosis, were merged and archived in order to form a clinical history database per patient for future comparisons.

The file transportation scenario employed through the server was selected for two reasons. Firstly like SCP-ECG, the e-SCP-ECG**^+^** standard is not very suitable for real-time transmission of biosignals (e.g., ECG) though it is very flexible and suitable for the storage of the data snapshots acquired. Secondly, it offers the possibility of switching between two RHMSs without loss of data. 

At the end of the five-month pilot period of the HTS, patients asked to complete a questionnaire about their experience on system's usage. Table 6 presents a subset of the used questionnaire and also the percentage given by patients on each category.

The participating doctors had also completed questionnaires relative to the systems response and usefulness. Table 7 presents a subset of the used questionnaire and also the percentage given by patients on each category.

## 6. Conclusions

This paper has introduced the extension of SCP-ECG protocol to e-SCP-ECG**^+^** protocol in order to satisfy health telemonitoring needs, as required by cooperating doctors during various telemedicine projects in the past [3, 4, 23]. The e-SCP-ECG**^+^** protocol adds the capability of handling more demographics data, extra vital signs such as the SPO_2_, the CO_2_, the NiBP, the Temp, and the pulse rate, and also data relative to allergies. The extensions are made both as additions to existing sections and as new sections for the SCP-ECG protocol following its structure. 

In order to evaluate the e-SCP-ECG**^+^**, we implemented the protocol as software components that integrated into a pilot HTS. Using the HTS we tested the ability of the above protocol to handle the collected information.

The e-SCP-ECG**^+^** protocol can be integrated into medical systems for administration of patient examination data. Alternatively, it can be used to form a service providing experts with the whole body of information about a patient's health status, leading to a more accurate diagnosis as well as more appropriate treatment. This protocol can also be used in Ubiquitous Health monitoring (UHM) systems managing the collected information. The Protocol's adaptive structure permits the management of data characterizing each particular event (incident), leading to shorter transmission and process times and minor storage space needs.

## Figures and Tables

**Table 1:** SCP-ECG protocol Data Structure.

Section	Type	Information Description
0	Required	Pointers to data areas in the record
1	Required	Header Information—Patient data/ECG acquisition data
2	Dependent	Huffman tables used in encoding of ECG data (if used)
3	Required	ECG lead definition
4	Optional	QRS locations (if reference beats are encoded)
5	Optional	Encoded reference beat data if reference beats are stored
6	Required	“Residual signal” after reference beat subtraction if reference beats are stored, or encoded rhythm data
7	Optional	Global measurements
8	Optional	Textual diagnosis from the “interpretive” device
9	Optional	Manufacturer specific diagnostic and over reading data from the “interpretive” device
10	Optional	Lead measurements results
11	Optional	Universal statement codes resulting from the interpretation

**Table 2:** Patient data/ECG acquisition data stored in Section-1.

tag	Contents
0	Last Name
1	First Name
2	Patient Identification Number
3	Second Last Name
10	Drugs
13	Diagnosis or Referral Indication
14	Acquiring Device Identification Number
15	Analyzing Device ID Number
16	Acquiring Institution Description
17	Analyzing Institution Description
18	Acquiring Department Description
19	Analyzing Department Description
20	Referring Physician
21	Latest Confirming Physician
22	Technician Description
23	Room Description
30	Free Text Field
31	ECG Sequence Number
35	Free-Text Medical History
255	

**Table 3:** New defined parameters stored in Section-1.

Tag	Length	Value (Parameter Data)		
200	length	Second patient ID (Text characters)		

201	2	Patient Nationality (Binary)		
		This has the following format:		
		*Byte *	*Contents*	
		1-2	binary: Nationality indication (ISO 3166.1) defined as:	
			0	Unspecified
			m*	n**

202	length	Patient Address (Text characters)		

203	length	Patient Phone Number (Text characters)		

204	1	Patient Religion (Binary)		
		This has the following format:		
		*Byte *	*Contents*	
		1	Binary: set equal to 255	
			0	Unspecified
			1	Atheist
			2	Baha'I
			3	Buddhism
			4	Christianity
			5	Confucianism
			6	Hinduism
			7	Islam
			8	Jainism
			9	Judaism
			10	Shinto
			11	Sikhism
			12	Daoism
			13	Zoroastrianism
			14–30	Reserved
			31–255	Manufacturer specific

205	length	Birth Place (Text characters)		

206	1	Patient Insurance (Binary)		
		This has the following format:		
		*Byte *	*Contents*	
		1-2	binary: Nationality indication (ISO 3166.1) defined as:	
			0	Unspecified

207	length	Memorial History (Free Text)		
		This field contains a text description of hereditary diseases.		

208	1	Blood Type (Binary)		
		This has the following format:		
		*Byte *	*Contents*	
		1	binary: set equal to 255	
			0	Unspecified
			1	A+
			2	A−
			3	B+
			4	B−
			5	AB+
			6	AB−
			7	O+
			8	O−
209	length	Profession (Free Text)		
		This field contains a text description of people profession.		

210	1	File access (Binary)		
		This has the following format:		
		*Byte *	*Contents*	
		1	Binary: type of file access	
			0	read/write
			1	read only
			2	Locked

211	length	Access restrictions (Binary)		
		This has the following format:		
		*Byte *	*Contents*	
		1	Binary: type of access restrictions	
			0	All file contents available to all medical specialties
			1	File contents available depended to medical specialties
			2	All file contents available to specific medical specialty
		2	Binary: if byte 1 has value different from 0 then this field contains the medical specialty. There is no limit on the number of specialties.	

212	length	SPO_2_ Machine ID Acquiring Device (Binary bytes and Text characters)		

213	length	NiBP Machine ID Acquiring Device (Binary bytes and Text characters)		

214	length	CO_2_ Machine ID Acquiring Device (Binary bytes and Text characters)		

215	length	GPS Machine ID Device (Binary bytes and Text characters)		

216	1	Operational mode (Binary)		
		This has the following format:		

		*Byte *	*Contents*	
		1	0	Basic
			1	Emergency

*m the numeric values and **n the description of the corresponding codes of countries in ISO 3166-1.

**Table 4:** Definition of Data Part of Section-204 (Allergy).

Tag	Length	Value (Parameter Data)				
1	3	Rhinitis (Binary)				
		This has the following format:				
		*Byte *	*Contents*			
		1	Binary: type of rhinitis			
			0: all year			
			1: seasonal			
			2: all year with Conjunctivitis			
			3: seasonal with Conjunctivitis			
		2	if seasonal, then appears on Month:			
			Bit 0	January	Set = Yes	Reset = No
			Bit 1	February	Set = Yes	Reset = No
			Bit 2	March	Set = Yes	Reset = No
			Bit 3	April	Set = Yes	Reset = No
			Bit 4	May	Set = Yes	Reset = No
			Bit 5	June	Set = Yes	Reset = No
			Bit 6	July	Set = Yes	Reset = No
			Bit 7	August	Set = Yes	Reset = No
		3	if seasonal, then appears on Month:			
			Bit 0	September	Set = Yes	Reset = No
			Bit 1	Octomber	Set = Yes	Reset = No
			Bit 2	November	Set = Yes	Reset = No
			Bit 3	December	Set = Yes	Reset = No

2	3	Asthma (Binary)				
		This has the following format:				
		*Byte *	*Contents*			
		1	Binary: type of rhinitis			
			0: all year			
			1: seasonal			
		2	if seasonal, then appears on Month:			
			Bit 0	January	Set = Yes	Reset = No
			Bit 1	February	Set = Yes	Reset = No
			Bit 2	March	Set = Yes	Reset = No
			Bit 3	April	Set = Yes	Reset = No
			Bit 4	May	Set = Yes	Reset = No
			Bit 5	June	Set = Yes	Reset = No
			Bit 6	July	Set = Yes	Reset = No
			Bit 7	August	Set = Yes	Reset = No
		3	if seasonal, then appears on Month:			
			Bit 0	September	Set = Yes	Reset = No
			Bit 1	Octomber	Set = Yes	Reset = No
			Bit 2	November	Set = Yes	Reset = No
			Bit 3	December	Set = Yes	Reset = No
4	length	Medical Allergy or Drug Allergy (Binary)				
		This has the following format:				
		*Byte *	*Contents*			
		1	binary: set equal to 255			
			0-Unspecified			
			1-Penicillin,			
			2-Sulfa antibiotics,			
			3-Allopurinol,			
			4-Seizure and anti-arrhythmia medications,			
			5-Nonsteroidal anti-inflammatory drugs (such as aspirin and ibuprofen),			
			6-Muscle relaxants,			
			7-Certain post-surgery fluids			
			100-Other			
		This field contains a list of the patient's allergy on foods. There is no limit of inputs. Each input shall be represented by one byte				

5	length	Food Allergy (Binary)				
		This has the following format:				
		*Byte *	*Contents*			
		1	binary: set equal to 255			
			0-Unspecified			
			1-Fish and shellfish			
			2-Peanuts			
			3-Tree nuts, such as walnuts			
			4-Eggs			
			5-Cow's Milk			
			6-Wheat			
			7-Soy			
			100-Other			
		This field contains a list of the patient's allergy on foods. There is no limit of inputs. Each input shall be represented by one byte				

6	length	Other Allergy (Text characters)				
		This field permits free text comments about other type of allergies.				

**Table 5:** Patient included in the evaluation-attributes.

Age	Patients Equipment		Malfunctions		
	Wearable	Conventional	Heart	Respiratory	Allergies
30–40	3	2	5	3	
41–50	9	6	15	7	2
51–60	2	5	7	1	3

**Table 6:** Patient's questionnaire subset.

Question on …	A	O	Θ
*System's characteristics*			
(1) System's reliability: error frequency	11	33	56
(2) Convenience on sensor placement	4	52	44
(3) Measurement device maintenance (charge,clearance…)	0	11	89

*Personal data*			
(1) Convenience on data input	4	19	78
(2) Are there data unethical or abusive?	0	15	85
(3) Are there data difficult to collect?	0	4	96

*System's learning*			
(1) Learning of device operation	0	30	70
(2) Learning of used software/application	0	30	70

*Communication with other systems—users*			
(1) Data transmission to the Data Reception Center	4	44	52
(2) Communication with Doctors in case of a health problem	0	7	93

*Difficulties during the usage*			
(1) Movement with the sensors in place	19	33	48
(2) Sensor slip during movement	7	19	74
(3) Limitations provided by sensors usage	22	30	48
(4) Irritations caused by sensor usage	74	22	4

*Total system's satisfaction*			
(1) System's reliability	4	22	74
(2) System's operation satisfaction	0	22	78

A: Negative (checked: −2, −1); O: Neutral (checked 0); Θ: Positive (checked: 1, 2).

**Table 7:** Doctor's questionnaire subset.

Question on …	A	O	Θ
*System's characteristics*			
(1) System's reliability: error frequency	0	0	100
(2) Workstation switching	0	0	100
(3) Incident presentation	0	33	67

*Data*			
(1) Data integrity	0	0	100
(2) Data resolution	0	0	100
(3) Ability to make diagnosis using the received data	0	0	100

*System's learning*			
(1) Learning of device operation	0	0	100
(2) Learning of used software/application	0	0	100

*Communication with other systems—users*			
(1) Data reception to the Data Reception Center	0	33	67
(2) Communication with Patients in case of a health problem	0	67	33

*Total system's satisfaction*			
(1) System's reliability	0	0	100
(2) System's operation satisfaction	0	0	100

A: Negative (checked: −2, −1); O: Neutral (checked 0); Θ: Positive (checked: 1, 2).

## Abbreviations

ADSL: Asymmetric Digital Subscriber Lines
DAS: Data Acquisition System
BAN: Body Area Network
C/S: Client/Server
DP: Data Part
ECG: Electrocardiograph
HSPA: High Speed Packet Access
HTS: Health Telemonitoring System
MM: Medical monitor
NiBP: Noninvasive blood pressure
P2P: Peer-to-peer
PDA: Personal digital assistant
RHMS: Remote Health Monitoring Station
SU: Storage Unit
TC: Telemedicine Center
Temp: Body temperature
UHM: Ubiquitous Health Monitoring
UA: User Authentication.
